# Intraoral image generation by progressive growing of generative adversarial network and evaluation of generated image quality by dentists

**DOI:** 10.1038/s41598-021-98043-3

**Published:** 2021-09-16

**Authors:** Kazuma Kokomoto, Rena Okawa, Kazuhiko Nakano, Kazunori Nozaki

**Affiliations:** 1 Division of Medical Informatics, Osaka University Dental Hospital, 1-8 Yamada-oka, Suita, Osaka 565-0871 Japan; 2 Department of Pediatric Dentistry, Osaka University Graduate School of Dentistry, 1-8 Yamada-oka, Suita, Osaka 565-0871 Japan

**Keywords:** Paediatric dentistry, Dental clinical teaching

## Abstract

Dentists need experience with clinical cases to practice specialized skills. However, the need to protect patient's private information limits their ability to utilize intraoral images obtained from clinical cases. In this study, since generating realistic images could make it possible to utilize intraoral images, progressive growing of generative adversarial networks are used to generate intraoral images. A total of 35,254 intraoral images were used as training data with resolutions of 128 × 128, 256 × 256, 512 × 512, and 1024 × 1024. The results of the training datasets with and without data augmentation were compared. The Sliced Wasserstein Distance was calculated to evaluate the generated images. Next, 50 real images and 50 generated images for each resolution were randomly selected and shuffled. 12 pediatric dentists were asked to observe these images and assess whether they were real or generated. The d prime of the 1024 × 1024 images was significantly higher than that of the other resolutions. In conclusion, generated intraoral images with resolutions of 512 × 512 or lower were so realistic that the dentists could not distinguish whether they were real or generated. This implies that the generated images can be used in dental education or data augmentation for deep learning, without privacy restrictions.

## Introduction

Medical workers should experience as many clinical cases as possible to develop their professional competence^1^. The oral examination is one of the most important techniques in decision-making in dentistry. Thus, dental clinicians need to study as many intraoral images as they can to increase their accuracy in oral examinations. This is especially true for pediatric dentistry since the oral environment of children drastically changes as children grow. In particular, it is important to understand the differences between primary teeth and adult teeth, the timing of tooth replacement, maxillofacial bone growth, or changes. in dental occlusion and alignment.

In recent years, deep learning using a convolutional neural network has been developed for computer vision and has been applied to medical image analysis. The generative versarial network (GAN) is an image generation method based on deep learning^2^, which is a class of unsupervised machine learning systems that consists of a generator network and discriminator network. In some reports, the generation of medical images using a GAN has been successful^3,4^. However, those studies have dealt with only grayscale X-ray images, and no evaluation of generated medical images by experts has been performed.

The need to protect a patient’s private information restricts the use of intraoral images obtained in clinical cases. For this reason, sharing as many intraoral images as possible among different hospitals is difficult^5^. Thus, increasing the number of images of similar clinical cases is essential for clinical dental education. To solve both, the nonreal intraoral images are expected to be generated by using GAN for education.

In this research, we describe the generation of full-color intraoral images using progressive growing of generative adversarial networks (PGGAN) and evaluate the generated intraoral images in terms of both quantity performance and visual quality assessed by pediatric dentists.

## Materials and methods

### Image datasets and preprocessing

The 35,254 intraoral images were used in this study as training data and were obtained from patients who received dental treatment between August 2008 and March 2019 at Osaka University Dental Hospital, Department of Pediatric Dentistry, Osaka, Japan. All images consisted of approximately 4000 × 3000 pixels and had an RGB color. These images included primary, mixed, and permanent dentition. There were various teeth conditions: healthy, caries, stain, composite resin restoration, metal inlay, stainless steel crown, space maintainer, orthodontic appliance, hypomineralization, etc. None of the images contained metadata, such as sex, age, tooth conditions, or diseases. Therefore, the exact number of each condition was unknown, but roughly speaking, the datasets contained a relatively high number of images that showed healthy teeth. All images were resized to 128 × 128, 256 × 256, 512 × 512, and 1024 × 1024 pixels and converted to the JPEG format. It is said that a small number of datasets can easily lead to overfitting, but the number of training data used in this study was limited, so data augmentation was adopted^6^. The results obtained with and without data augmentation were compared. Each resized image was randomly augmented five times using the ImageDataGenerator function that is built into Keras^7^, whose parameters were rotation_range = 20, width_shift_range = 0.1, height_shift_range = 0.1, shear_range = 0.1, zoom_range = 0.1, horizontal_flip = True, and fill_mode = 'nearest'. All computations were performed on a Ubuntu 18.04 desktop computer with one NVIDIA TITAN RTX.

### Network architecture

**Figure 1:**
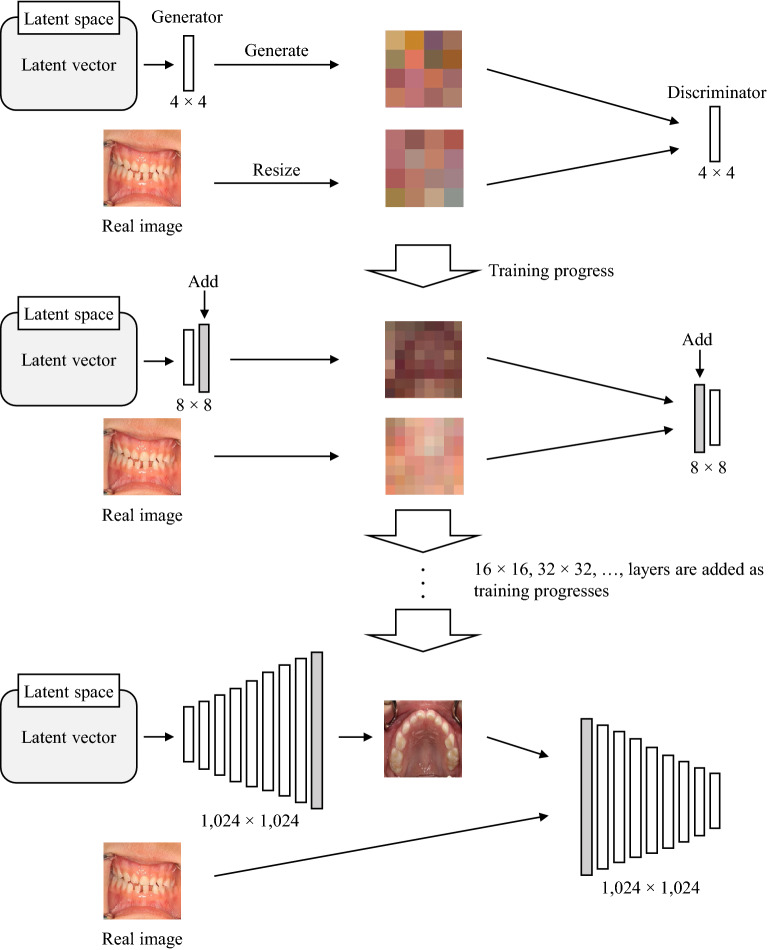
The architecture of progressive growing generative adversarial networks (PGGAN).

A GAN is a framework that consists of two adversarial neural networks based on deep learning. One is a generative network (*G*), and the other is a discriminative network (*D*). *D* is trained to distinguish between true and generated samples, and *G* is trained to fool *D* with its generated samples. This framework corresponds to a strategy for making decisions to minimize possible damage. As a result of this learning, *G* can output realistic images. However, many types of GANs have failed to generate high-resolution images, mainly generating 256 × 256 or lower resolution images, until the PGGANs, which has made it possible to generate 1024 × 1024 high-resolution images from latent vectors with improved quality, stability, and variation^8^. The PGGAN starts training with low resolution at first and progressively increases the resolution by adding new layers to both the generator and discriminator (Fig. 1). We trained the PGGAN using resized real intraoral images of 128 × 128, 256 × 256, 512 × 512, and 1024 × 1024 resolution with and without data augmentation, and the images generated by *G* for each resolution were evaluated.

### Evaluation

The visual quality of intraoral images is an important factor. 50 generated images that seemed to be real at first glance were manually selected by one pediatric dentist, and 50 real images were added, followed by random shuffling and reordering. All chosen images were different for all resolutions of 128 × 128, 256 × 256, 512 × 512, and 1024 × 1024, which resulted in a total of 400 images. Since paper resumes were often used in education, all images were printed on one side using a Canon LBP7200 printer instead of digital display. The paper used for printing was ASKUL Multipaper Super White J (thickness 0.09 mm, basis weight 68 g/m^2^, whiteness level 92%). Evaluation of these images was performed by 12 pediatric dentists who belonged to the Department of Pediatric Dentistry, Osaka University Graduate School of Dentistry, and consisted of 5 men and 7 women. 10 of them had less than 6 years of clinical experience, and the remaining 2 had more than 10 years of clinical experience.

These 12 pediatric dentists were asked to observe randomly shuffled images that consisted of 50 real images and 50 generated images for each resolution described above, but the ratio of real and generated images was not told to the dentists and asked to assess a total of 400 images whether each image was real or generated. When the given image was determined to be generated, the discriminative parts of each image were marked by evaluators. The elements for discrimination of real or generated were categorized, and the number of times each element was used for discrimination was counted. Each dentist evaluated the images alone without consulting with other dentists, and all experiments were performed in the same room under the same lighting conditions. Because there is a probability of randomly answering in a limited time, the evaluation time was set to be unlimited so that each dentist could assess images carefully.

Since there were individual differences in 12 dentists, human observers were not ideal, and human biases should be taken into account. Instead of the accuracy calculated by the confusion matrix, the d prime of each dentist for each resolution was calculated based on the signal detection theory^9^. The d prime is one of the metrics of an individual’s ability to discriminate given signals and is not affected by response biases. The larger the d prime is, the easier it is to discriminate whether the given images are real or generated.

The d prime for each resolution was analyzed using an Analysis of Variance (ANOVA) and a multiple comparison with Tukey’s honestly significant differences test (TukeyHSD test) after checking the normality of each answer with the Shapiro–Wilk test and checking the equal variances with Bartlett’s test. Statistical power and effect size (Cohen’s f) were also calculated from the results of the ANOVA^10^. The discriminative parts marked by dentists were categorized, and the differences between each resolution in each category were analyzed using the Kruskal–Wallis test. Statistical significance was set at p < 0.05.

Some metrics have been proposed to evaluate the quantitative performance of generated images. In this study, we used Sliced Wasserstein Distance (SWD) metrics, which were proposed in the original PGGAN article^8^. The SWD was calculated for each level of the Laplacian pyramids^11^ of the randomly sampled 16,384 images, and the average SWD of each Laplacian pyramid was calculated. A small SWD value indicates that the true images and generated images seemed to be similar in both appearance and variation, so the weightparameters of *G* that showed the lowest SWD values were used for generating images.

### Ethics

The Ethics Committee of Osaka University Graduate School of Dentistry approved this study (approval: R1-E29) and the need for informed consent was waived. It is confirmed that all methods in this study were performed in accordance with the Act on the Protection of Personal Information and Ethical Guidelines for Medical and Health Research Involving Human Subjects.

## Results

### Visual quality evaluation

**Figure 2:**
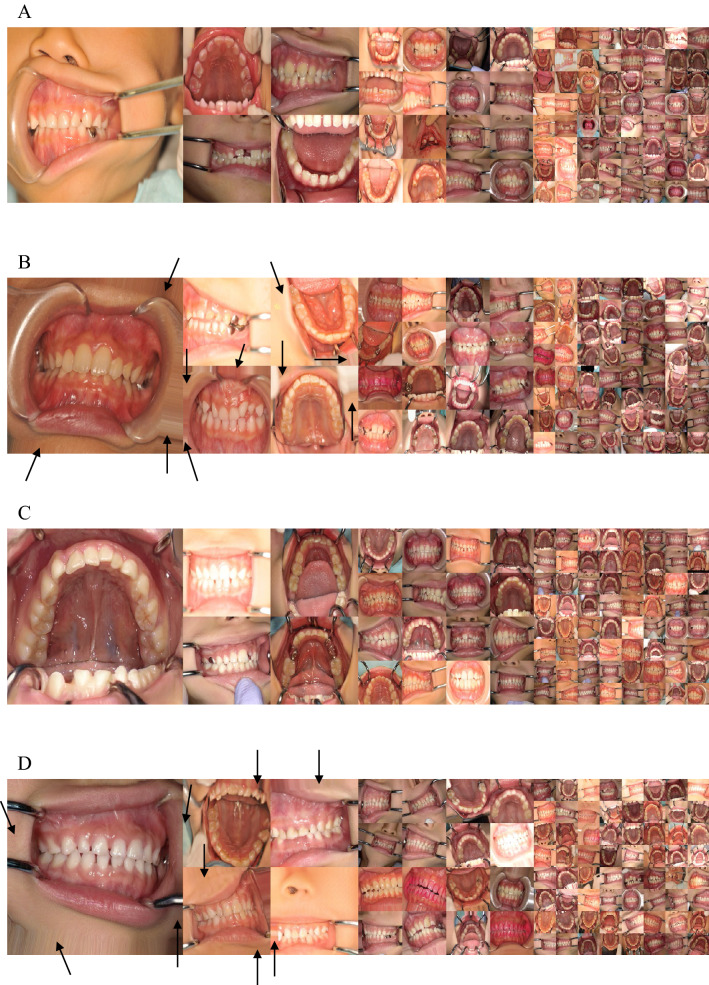
Examples of images used in this study with a resolution of 1024 × 1024, 512 × 512, 256 × 256, and 128 × 128. Arrows indicate the examples where the boundaries of the training images with data augmentation are filled with the closest pixel value and repeated for all the empty values, which is the effect of the fill_mode of ImageDataGenerator. (**A**) Real images used for training without data augmentation. (**B**) Real images used for training with data augmentation. (**C**) Generated images trained with real images that were not augmented. (**D**) Generated images trained with real images that were augmented.

The visual quality was compared between the real and generated images (Fig. 2). When compared to the original images (Fig. 2A), the boundaries of the training images with data augmentation are filled with the closest pixel value, and this is repeated for all the empty values, which is the effect of the fill_mode of ImageDataGenerator (Fig. 2B). The boundaries of the generated images trained without augmented real images were clear (Fig. 2C). However, the boundaries of the trained with augmented real images are filled with the closest pixel value (Fig. 2D), which is the same as that in Fig. 2B.

The generated images were evaluated by 12 pediatric dentists for visual quality assessment. The images generated by PGGAN trained with augmented real images were not evaluated because the boundaries of the images were clearly different from the boundaries of the real images as described above, so only the images generated by PGGAN trained without augmented real images were evaluated.

**Figure 3:**
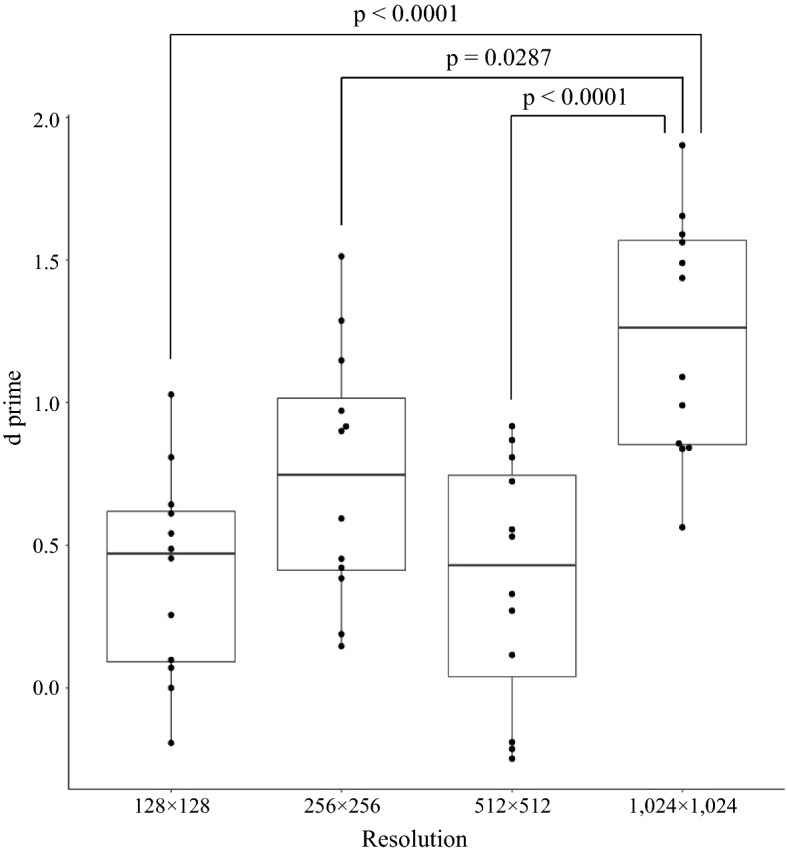
Boxplot of d prime for each resolution. The black dots indicate the raw data of the 12 dentists. The d prime of 1024 × 1024 was significantly higher (TukeyHSD test).

The d prime was calculated for each resolution using the responses of the 12 pediatric dentists, and statistical analysis was performed. The responses of each resolution were tested for normality using the Shapiro–Wilk test. The p-value for resolutions of 128 × 128, 256 × 256, 512 × 512, and 1024 × 1024 were 0.9627, 0.6184, 0.2073, and 0.4271, respectively. As a result, each answer was considered to have a normal distribution. Bartlett’s test was then performed for homogeneity of variance. The p-value was 0.9152, which means that each answer was considered to be from populations with equal variances (Table 1). Therefore, ANOVA and Tukey’s HSD tests were used for multiple comparisons (Tables 2, 3). The statistical power was calculated to be 0.99, which indicated that 12 dentists were sufficient to perform our study. The d prime of 1024 × 1024 resolution was significantly higher than that of 128 × 128, 256 × 256, and 512 × 512 resolutions No statistically significant differences were observed with the other combinations (Fig. 3, Table 3).

**Table 1:** Check of normality of each answer of d prime with the Shapiro–Wilk test and the check of equal variances with Bartlett’s test.

Resolution	Shapiro–Wilk test	Bartlett’s test
128 × 128	0.9627	0.9152
256 × 256	0.6184	
512 × 512	0.2073	
1024 × 1024	0.4271	

All p-values were more than 0.05, which indicated that all answers were considered to have normal distribution and equal variances.

**Table 2:** ANOVA of the 12 dentists’ d prime for each resolution.

	Degree of freedom	F value	p value	Cohen’s f
Resolutions	3	11.25	< 0.0001*	0.88
Residuals	44			

The statistical power was 0.99, which indicated that 12 dentists were enough to perform our study.

* represents p < 0.05.

**Table 3:** TukeyHSD of the 12 dentists’ d prime for each resolution with 95% confidence level.

Comparison	p value
128 × 128–256 × 256	0.1941
128 × 128–512 × 512	0.9983
128 × 128–1024 × 1024	< 0.0001*
256 × 256–512 × 512	0.1418
256 × 256–1024 × 1024	0.0287*
512 × 512–1024 × 1024	< 0.0001*

The d prime of 1024 × 1024 resolution was significantly higher than that of 128 × 128, 256 × 256, and 512 × 512 resolutions.

* represents p < 0.05.

**Figure 4:**
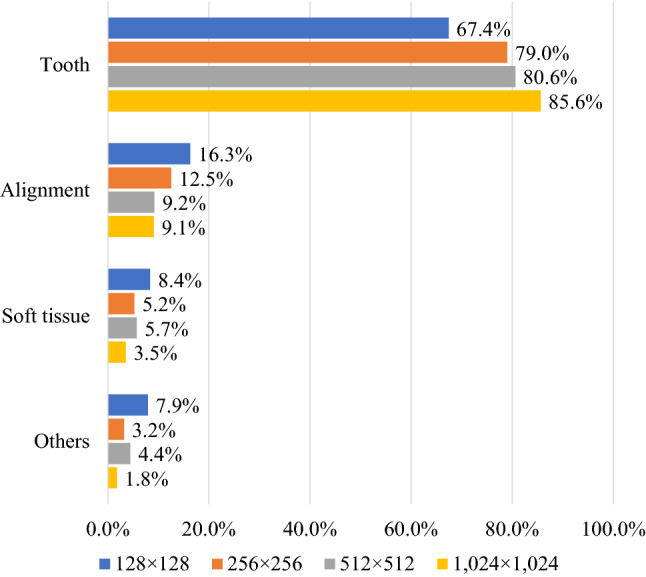
Ratio of each element used for criteria to assess whether the given image is real or generated. Most of the dentists used teeth to discriminate images.

The parts of the images the dentists looked at to determine whether they were real or generated were categorized into four factors: tooth, alignment, soft tissues, and others. Most of the dentists used teeth as the assessment criteria (Fig. 4). For each factor, the Kruskal–Wallis test showed no significant differences between the resolutions (Table 4).

**Table 4:** Kruskal–Wallis test of the differences between each resolution.

	Tooth	Alignment	Soft tissue	Others
p value	0.5004	0.2874	0.1435	0.1783

No significant differences have been found in the number of counts of each discriminative part of images.

### Quantitative evaluation

Table 5 shows the SWD values for each resolution. With resolutions of 128 × 128, 256 × 256, and 512 × 512, the SWD was reduced by data augmentation of the training data, which means that the quality of the generated image was improved. On the other hand, the SWD value increased with a resolution of 1024 × 1024, which means that data augmentation has shown negative improvement in the quality of the generated image.

**Table 5:** SWD values of each resolution with and without data augmentation.

Resolutions	Augmentation	
	Not used	Used
128 × 128	5.77	4.25
256 × 256	5.49	4.68
512 × 512	5.58	5.45
1024 × 1024	9.23	12.57

A small value of SWD indicates that the true images and generated images seem to be similar in both appearance and variation.

## Discussion

### Quality of generated oral images

In this study, we present synthetic intraoral images using PGGAN. When the dentists evaluated the visual quality of the generated images, only the resolution of 1024 × 1024 showed a significantly higher d prime (Fig. 3). Since 1024 × 1024 is a higher resolution, the generated image may have been noticeably rougher than the other images, which led to easy discrimination by the dentists. This is considered to be consistent with the fact that the SWD value of 1024 × 1024 is only higher than that of the other resolutions (Table 5). There is a possibility that artifacts in the generated images may be invisibly small and hidden at low resolutions. However, even if a quantitative metric such as SWD provides the best results, not all generated images can be used for education. It is desirable to use the generated images after filtering by experts, so both quantitative and qualitative evaluations were performed in our study. If the pediatric dentists cannot recognize the artifacts, the generated images are considered to be acceptable for educational materials. In other words, it is considered that the generated intraoral images with lower resolutions are so good that the dentist cannot distinguish whether they are real or generated images.

The reason why the SWD value of 1024 × 1024 has been increased by data augmentation is considered to be the filled pixels on boundaries. Although the quality of the image deteriorates owing to the filled pixels of boundaries, the filled area is small at 512 × 512 or lower resolutions, and it is considered that the increase in image variation by data augmentation contributes to a decrease in the SWD value. On the other hand, at 1024 × 1024 resolution, because the filled area is larger than that of lower resolutions, it is considered that the influence of deterioration of image quality is more affected than the increase in image variation, which leads to an increase in the SWD value. At 512 × 512 or lower resolutions, data augmentation decreased the SWD values. If the filled pixels of the image boundaries were cut out, the remaining central images could be of better quality than the images generated by PGGAN trained without dataaugmentation.

**Figure 5:**
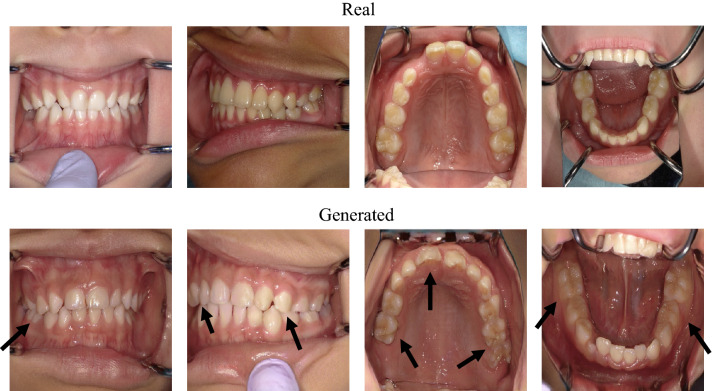
Comparison between real and generated intraoral images. Arrows indicate points of strange teeth. Tooth alignment and soft tissues in both images are similar.

Focusing on the teeth is one of the key factors in distinguishing whether an image is real or generated (Fig. 4). Some teeth in generated images look strange compared with natural teeth; however, teeth alignment or soft tissue, such as the tongue, lips, and nose, look realistic enough (Fig. 5). This is because, for primary and permanent teeth, there are many types of anatomical shapes, colors, fissures, cusps, and outlines than types of alignments or soft tissues for each person; hence, PGGAN cannot learn and generate tooth features with our limited dataset. So people who use the generated images need to examine whether the images are misleading. Our results indicate that the teeth were critical for detecting the generated images. However, this does not mean that other factors are not important. It should be noted that alignment, soft tissue, or any other intraoral information is equally important in both dental education and dental treatment.

### Research limitation

The efficiency of generating realistic images using PGGAN markedly depends on the number of learned images. Even if dentists often take intraoral images in daily clinical treatment, only approximately 35,000 intraoral images could have been stored between 2008 and 2019. The original PGGAN was trained with the CelebA dataset, which consists of approximately 200,000 face images^12^, and has achieved significant performance. Thus, our results may not utilize the maximum efficiency of PGGAN. In addition, our datasets contained a relatively higher number of images that showed healthy teeth than that of diseases, and there was also a tendency in PGGAN to generate healthy images compared to disease. If we were able to add images of diseases and adjust the class imbalance, we could have improved the performance of PGGAN. However, evaluators did not know the class imbalance of the datasets, and it is not affect the discrimination of images by dentists.

When dentists try to take intraoral images, cheek retractors and intraoral mirrors are placed in the mouth, and the camera axis and patient’s head should be parallel^13^. However, since most children cannot stay still and cannot open their mouth enough for an intraoral mirror to be inserted, it is difficult to obtain unified intraoral images. In addition, it is impossible to correct inappropriate intraoral images due to perspective and distortion, even if photo editing software is used. For these reasons, a large-scale unified and class-balanced public dental dataset needs to be constructed to enable the clinical application of deep learning with meaningful performance.

In order to challenge the generation of 1024 × 1024 with higher quality than PGGAN, another generative deep learning method is needed. For example, StyleGAN or StyleGAN2 can generate better images than PGGAN^14,15^. However, these new generative networks require a large amount of machine power. It has been reported that the training time of 1024 × 1024 resolution with StyleGAN is approximately one week, and that of StyleGAN2 is 9 days on NVIDIA DGX-1 with 8 Tesla V100 GPUs. If we try to perform these networks on our one TITAN RTX, it is estimated that the training time takes more than two or three months. On the other hand, there are few differences between 512 × 512 and 1024 × 1024 resolutions in dental diagnosis or examination, because dental conditions such as tooth shape, tooth color, tooth arrangement, caries, and metal crowns can be recognized with 512 × 512 resolution images. In addition, the training time of 512 × 512 resolution with PGGAN is approximately 8 days on TITAN RTX, which is a more acceptable time than StyleGAN. It is considered that PGGAN has sufficient ability to generate 512 × 512 or lower resolution intraoral images with reasonable machine power and time compared with new generative methods that require a large amount of computational resources.

It is critical to perform experiments that require domain-specific knowledge; however, the number of specialists is generally small and limited compared to general dentists. The 12 pediatric dentists in this study were almost all the pediatric dentists in our hospital, and it was almost the limit that cannot be increased further. Therefore, it is important to know in advance the number of specialists required for further studies. The number of participants that satisfied the statistical power of 0.9, was calculated to be 5.66 with our Cohen’s f (Table 2)^10^. If at least six specialists are recruited, it is possible that an experiment similar to our study can be performed in other medical fields.

### Future direction and applications

**Figure 6:**
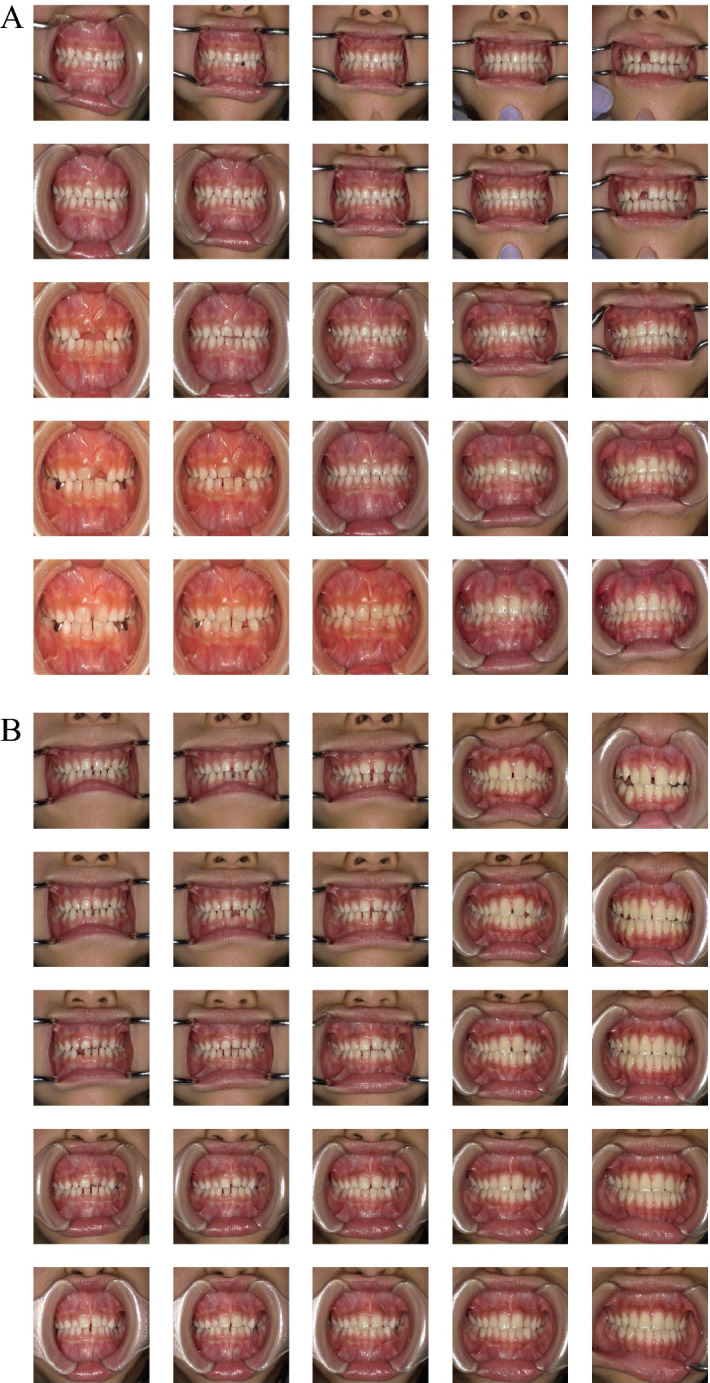
An example of exploring latent space. The images generated by PGGAN are gradually changing between one end and the other, from primary dentition to permanent dentition. One Supplementary Movie which shows intraoral image generation movie associated with this figure can be found in the online version.

There is a possibility that many types of images can be generated by changing the value of the latent vector. For example, image morphing can be performed by exploring the latent space^16^. In our study, morphing of intraoral images can be achieved by linearly interpolating the latent vectors that generate images of the primary dentition, mixed dentition, and permanent dentition (Fig. 6). It seems that images change transitionally between one end and the other as if the color changes with gradation. If arbitrary images can be generated with various types of images, PGGAN is a useful tool for dental education or for explaining materials to patients. Supplementary data showing intraoral image-generation movies can be found in the online version.

Another advantage of using generated intraoral images is their ease of use. Because the images can be generated not to include private information, researchers or educators do not need to take care of individual information and can feel free to use the generated intraoral images.

In this study, we used the latent vector as input data for the PGGAN to generate images. If the PGGAN architecture is modified and the real images can be used for input data instead of the latent vector, such as pix2pix^17^ or CycleGAN^18^, there is a possibility that intraoral images of the future can be generated and predicted from current intraoral images. If children could be informed that their future teeth alignment is likely to be bad, early orthodontic treatment could be recommended, which would save money and time. Also, if intraoral images of the future could be generated that show the differences between getting treatment and not getting it, dentists would be better able to recommend dental treatment to their patients. Patients could be motivated to think about their oral health and be encouraged to brush their teeth carefully at home by being shown images of a future with periodontitis or tooth loss caused by not taking care of their teeth.

Another way of applying PGGAN is to use the generated images for data augmentation in deep learning. When we perform a deep learning project, many medical images for training data are required to achieve good performance. It is said that a small amount of training data can easily lead to overfitting in the deep learning model, so data augmentation of training images, such as translation, rotation, zoom, and contrast, is commonly used to reduce overfitting^6^. In addition to such data augmentation, it is expected that the generated realistic and varied images are used for deep learning as a method of data augmentation. It has been reported that the accuracy of medical image recognition based on deep learning has been improved by adding artificial images generated by GAN to the training data^19–21^. In the future, our findings may contribute to improving the performance of deep learning related to intraoral images by using generated images, since generated images are so realistic that pediatric dentists cannot distinguish which image is real or generated.

## Conclusion

In this study, we present the intraoral images generated by PGGAN where resolutions of 512 × 512 or lower were so realistic that dentists could not distinguish whether they were real or generated. This implies that the generated intraoral images can be used as educational materials or for data augmentation for deep learning that is free from privacy restrictions.

## Supplementary Information

Supplementary Legends.

Supplementary Movie 1.
