# Supplementary material for: Intraoral image generation by progressive growing of generative adversarial network and evaluation of generated image quality by dentists
Source: Sci Rep. 2021 Sep 16;11:18517. doi: 10.1038/s41598-021-98043-3 (PMC8445945; doi:10.1038/s41598-021-98043-3)
Supplement: Supplementary file 1 — Supplementary Legends. [file 41598_2021_98043_MOESM1_ESM.docx]

**SUPPLEMENTARY DATA**

One supplementary data “Supplemental.mp4” which shows intraoral image generation movie associated with this article can be found in the online version.
